# Receptor-Like Cytoplasmic Kinase STK Confers Salt Tolerance in Rice

**DOI:** 10.1186/s12284-023-00637-0

**Published:** 2023-04-21

**Authors:** Yanbiao Zhou, Zhihui Zhang, Xinhui Zhao, Lan Liu, Qianying Tang, Jun Fu, Xiaodan Tang, Runqiu Yang, Jianzhong Lin, Xuanming Liu, Yuanzhu Yang

**Affiliations:** 1 State Key Laboratory of Hybrid Rice, Hunan Hybrid Rice Research Center, Changsha, 410125 Hunan China; 2 Key Laboratory of Southern Rice Innovation and Improvement, Ministry of Agriculture and Rural Affairs, Yuan Longping High-Tech Agriculture Co., Ltd., Changsha, 410001 Hunan China; 3 College of Life Sciences, South China Agricultural University, Guangzhou, 510642 China; 4 College of Plant Science and Technology, Huazhong Agricultural University, Wuhan, 430070 Hubei China; 5 College of Agronomy, Hunan Agricultural University, Changsha, 410128 Hunan China; 6 College of Life Sciences, Hunan Normal University, Changsha, 410081 Hunan China; 7 Hunan Province Key Laboratory of Plant Functional Genomics and Developmental Regulation, College of Biology, Hunan University, Changsha, 410082 Hunan China

**Keywords:** *STK*, Salt stress, Rice, ROS scavenging, ABA

## Abstract

**Background:**

Soil salinization is a major abiotic environmental stress factor threatening crop production throughout the world. Salt stress drastically affects the growth, development, and grain yield of rice (*Oryza sativa* L.), and the improvement of rice tolerance to salt stress is a desirable approach for meeting increasing food demand. Receptor-like cytoplasmic kinases (RLCKs) play essential roles in plant growth, development and responses to environmental stresses. However, little is known about their functions in salt stress. Previous reports have demonstrated that overexpression of an RLCK gene *SALT TOLERANCE KINASE* (*STK*) enhances salt tolerance in rice, and that *STK* may regulate the expression of GST (Glutathione S-transferase) genes.

**Results:**

The expression of *STK* was rapidly induced by ABA. *STK* was highest expressed in the stem at the heading stage. STK was localized at the plasma membrane. Overexpression of *STK* in rice increased tolerance to salt stress and oxidative stress by increasing ROS scavenging ability and ABA sensitivity. In contrast, CRISPR/Cas9-mediated knockout of *STK* increased the sensitivity of rice to salt stress and oxidative stress. Transcriptome sequencing analysis suggested that *STK* increased the expression of GST genes (*LOC_Os03g17480*, *LOC_Os10g38140* and *LOC_Os10g38710*) under salt stress. Reverse transcription quantitative PCR (RT-qPCR) suggested that four stress-related genes may be regulated by *STK* including *OsABAR1*, *Os3BGlu6*, *OSBZ8* and *OsSIK1*.

**Conclusions:**

These findings suggest that *STK* plays a positive regulatory role in salt stress tolerance by inducing antioxidant defense and associated with the ABA signaling pathway in rice.

**Supplementary Information:**

The online version contains supplementary material available at 10.1186/s12284-023-00637-0.

## Background

Salinity is one of the major abiotic stresses which influences plant growth and can lead to severe crop production losses (Chen et al. 2021). Approximately 46 million hectares of farmland in the world is subject to salt conditions, and this proportion is increasing every year due to inappropriate crop irrigation, over fertilization and excessive plowing, as well as natural reasons, such as salt intrusion into coastal zones caused by rising sea levels (Liu et al. 2021). Understanding the mechanisms of salt tolerance in rice will allow improving their yield and quality in areas subject to salt stress.

Reactive oxygen species (ROS) in plants include superoxide anion (O_2_), singlet oxygen (^1^O_2_), hydroxyl radical (OH^−^), and hydrogen peroxide (H_2_O_2_), which serve as key signaling molecules in many biological processes, involving biotic and abiotic stress tolerance (Mittler et al. 2011; Schippers et al. 2012; Schmidt et al. 2013). ROS production is enhanced in plants suffering from various abiotic stresses, such as salt, drought, and temperature (Mittler et al. 2004). Low ROS concentrations can function as a signal to activate salt stress responses, but excess ROS can result in oxidative damage to cellular membranes and other cellular components, which ultimately leads to cell death (Apel and Hirt 2004). To balance ROS production and destruction, plants have evolved an antioxidant system that involves both antioxidant enzymes, such as ascorbate peroxidase (APX), superoxide dismutase (SOD), catalase (CAT), glutathione S-transferase (GST), glutathione reductase (GR), and glutathione peroxidase (GPX), and antioxidant compounds, such as ascorbate (ASC) and glutathione (GSH), to scavenge ROS (Mittler 2002; Apel and Hirt 2004; Foyer and Noctor 2011). Identifying genes modulating the activities of antioxidant enzymes and antioxidant compounds content will be essential for improving plant stress tolerance. Overexpression of a receptor-like kinase gene *OsSIK1* in rice enhanced plant tolerance to drought and salt stress through activating the antioxidant enzymes to scavenging and detoxification of ROS (Ouyang et al. 2010). The genes encoding ROS scavenging enzymes were down-regulated in the *ospp18* mutant, and the mutant showed reduced activities of ROS scavenging enzymes and increased sensitivity to oxidative stresses (You et al. 2014). A loss of function of *CSN5B* enhances tolerance to salt by increasing ascorbic acid (AsA) synthesis (Wang et al. 2013).

Receptor-like kinases (RLKs) are transmembrane proteins with an extracellular domain, a transmembrane domain, and an intracellular kinase domain (Torii 2000). RLKs contain a large plant protein family with over 610 members in Arabidopsis and over 1131 in rice (Shiu et al. 2004). However, there are plant-specific RLKs without an extracellular domain and these RLKs contain only the transmembrane domain and intracellular kinase domain or only an intracellular kinase domain (Vij et al. 2008). This group are referred to as receptor-like cytoplasmic kinases (RLCKs). There are 200 genes encoding RLCKs in Arabidopsis and 379 in rice (Jurca et al. 2008; Vij et al. 2008). Eighty-two OsRLCKs are predicted to participate in abiotic stresses in rice (Vij et al. 2008). However, only a few RLCKs have been functionally characterized to be involved in abiotic stress response. A rice receptor-like cytoplasmic kinase, GUDK, was reported to phosphorylate OsAP37 and activate stress-induced gene expression, which improve drought tolerance and grain yield (Ramegowda et al. 2014). Cold-responsive protein kinase 1 (CRPK1) was reported to negatively regulate cold stress by phosphorylating 14-3-3 proteins, which interact with C-repeat binding factor (CBF) proteins and promote their degradation in the nucleus (Liu et al. 2017). A novel rice RLCK, STRK1, improves salt and oxidative tolerance by phosphorylating and activating CatC and thereby regulating H_2_O_2_ homeostasis (Zhou et al. 2018). Overexpression of *OsRLCK241* improved tolerance of rice plants to salt and drought stresses with improved ROS detoxification and altered expression of stress-responsive genes (Zhang et al. 2021).

Previously, we have identified RLCK genes from rice and examined their expression patterns in response to salt stress (Zhou et al. 2018). One of the salt-responsive RLCK genes, *SALT TOLERANCE KINASE* (*STK*), which encodes a putative receptor-like cytoplasmic kinase was selected for further analysis. This gene was induced by salt stress. To explore the potential function of *STK* in the salt stress response, transgenic plants were created with the overexpression of *STK* and knockout of *STK* in kitaake, respectively. Overexpression of *STK* improved tolerance to salt and oxidative stress through the enhanced activity of ROS scavenging enzyme GST and regulating the expression of multiple stress-related genes, whereas the *STK* knockout mutants increased salt sensitivity.

## Results

### The Expression Profiles and Subcellular Localization of *STK*

**Fig. 1:**
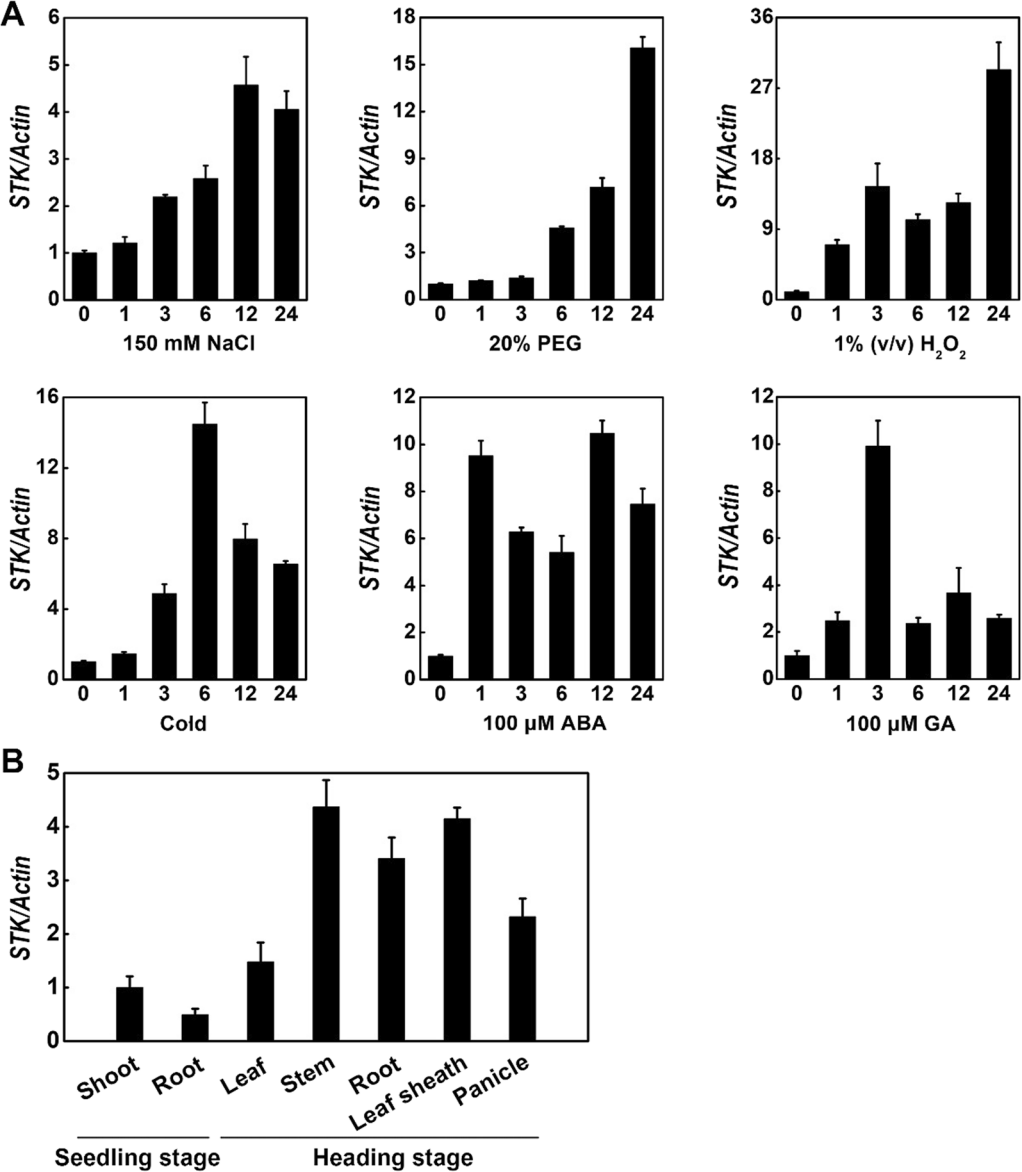
Expression patterns of *STK*. **A** Expression profiles of *STK* in Kitaake rice seedlings in response to salt, drought, H_2_O_2_, cold, ABA and GA treatments using RT-qPCR. **B** Expression of *STK* in various tissues of rice cultivar Kitaake at different stages using RT-qPCR. Data are means ± SD (n = 3)

To elucidate important factors for mediating salt stress signaling, we identified RLCK genes whose expression was induced by salt stress from our previous study (Zhou et al. 2018). Among these genes, we selected a salt stress-responsive RLCK gene named *SALT TOLERANCE KINASE* (*STK*), which belongs to the RLCK-VIIa sub-family (Gao and Xue 2012). To examine the physiological function of *STK* under abiotic stress conditions, the expression pattern of *STK* was examined in leaves of 3-week-old plants (*Oryza. sativa* cv Kitaake) in response to various abiotic stresses and treatments by reverse transcription quantitative PCR (RT-qPCR). Expression of *STK* was apparently induced under six treatments, including NaCl (150 mM), PEG (20%), H_2_O_2_ (1%), Cold (4 °C), ABA (100 μM) and GA (100 μM). ABA treatment led to earlier expression peak of *STK* than other treatments (Fig. 1A). These results suggested that *STK* is involved in responses to multiple abiotic stresses and treatments.

To investigate the tissue-specific expression of *STK* in the rice cultivar kitaake, the expression of *STK* was measured by RT-qPCR in various rice organs during the seedling and heading stages. The expression level of *STK* was higher in the shoot than in the root at the seedling stage (Fig. 1B). In addition, *STK* was expressed in all the tissues at the heading stage, with the highest level of expression in the stem, and the lowest level of expression in the leaf (Fig. 1B).

**Fig. 2:**
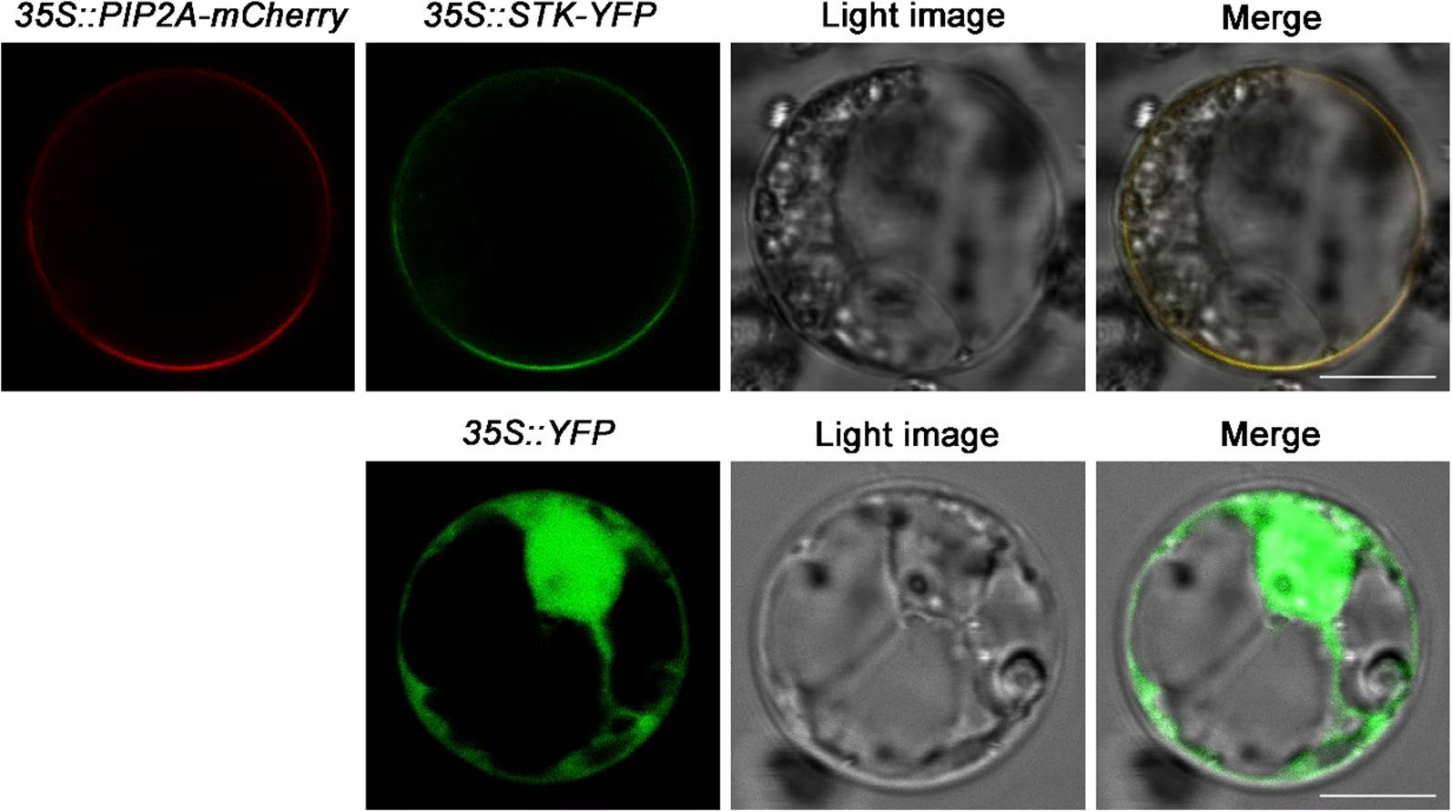
Subcellular localization of STK in rice protoplast. *35S::STK-YFP* and *35S::PIP2A-mCherry* were cotransformed into rice etiolated shoot protoplasts. *35S::YFP* was transformed as control. Bar = 10 μm

To determine the subcellular localization of STK, the full-length *STK* coding region was fused in frame to the yellow fluorescent protein (YFP) marker gene under the control of cauliflower mosaic virus 35S promoter. Rice protoplasts prepared from an etiolated shoot were cotransformed with *35S::STK-YFP* and *35::PIP2A-mCherry* by polyethylene glycol treatment. *PIP2A* was used as a marker since it has been reported as a plasma membrane aquaporin (Nelson et al. 2007). As shown in Fig. 2, the STK-YFP fusion protein was located in the plasma membrane; the plasma membrane localization was confirmed by its colocalization with the red fluorescence protein (mCherry)-fused plasma membrane protein PIP2A, indicating that STK is a plasma membrane protein.

### STK Positively Regulates Salt Tolerance in Rice

To evaluate the biological role of *STK*, transgenic rice plants with *STK* overexpressed under the control of the 35S promoter (*STK*-OE) and knockout by CRISPR/Cas9 system (*STK*-KO) were generated. One target site within the second exon was designed and an expression vector was constructed according to previously described CRSPR/Cas9 vector (Additional file 1: Fig. S1A, B) (Zhou et al. 2022). Two homozygous mutants (KO1 and KO3) were identified by sequencing (Additional file 1: Fig. S1C). KO1 contains an A insertion and KO3 contains a G deletion in the CDS, which caused a frameshift mutation (Additional file 1: Fig. S1D). Two independent *STK*-OE transgenic lines (OE3 and OE5; Additional file 1: Fig. S2) and two *STK*-KO lines (KO1 and KO3; Additional file 1: Fig. S1) of *STK* were selected for further analysis.

**Fig. 3:**
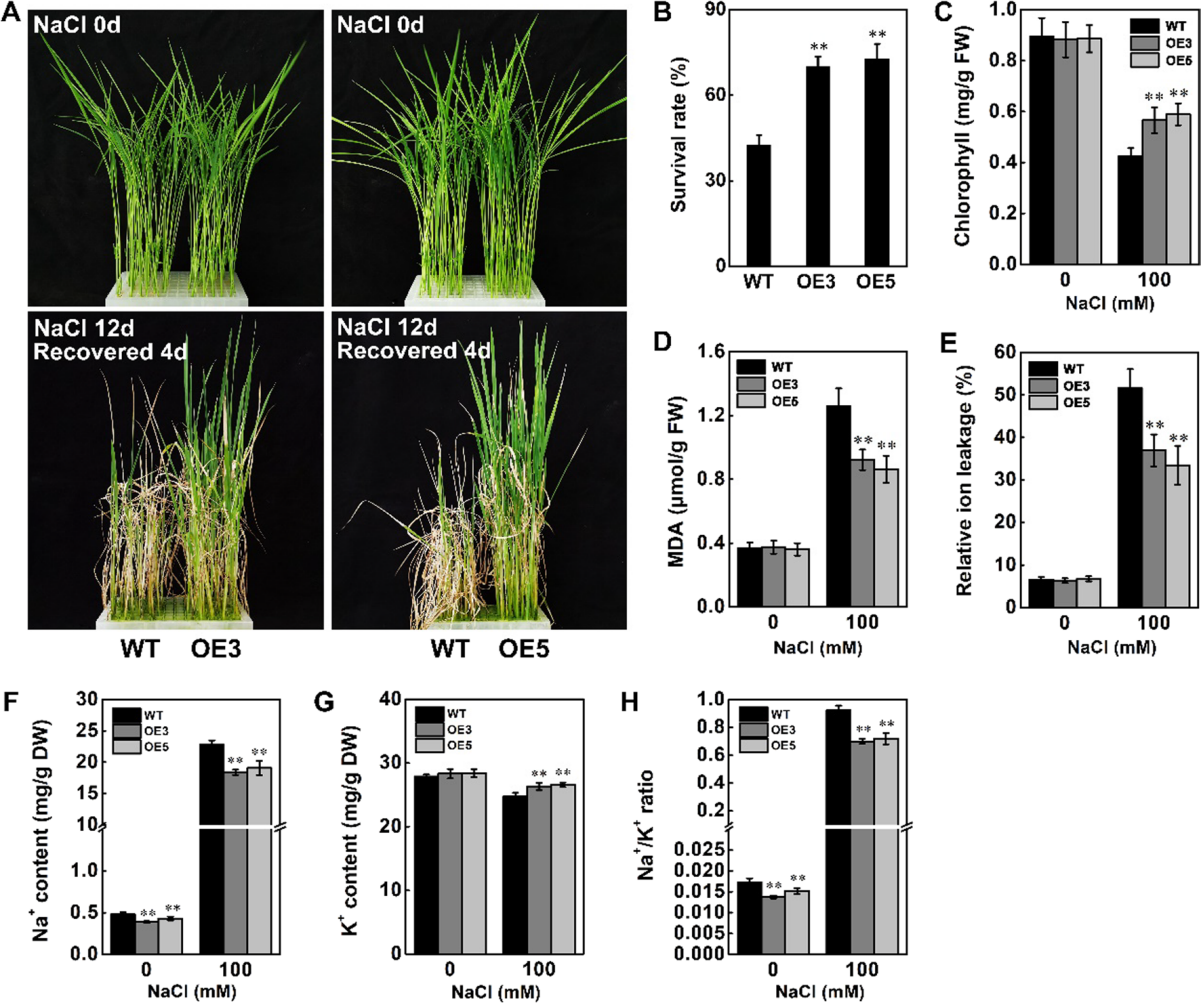
Overexpression of *STK* increased salt tolerance in rice. **A** Phenotype of WT, OE3 and OE5 rice plants under salt stress at seedling stage. For *STK*-OE transgenic plants, 15-d-old seedlings were treated with 140 mM NaCl for 12 d and recovery 4 d. **B** Survival rates of WT and transgenic plants in (A) after 4 d of recovery. **C** Chlorophyll content in leaves of 15-d-old plants treated with 100 mM NaCl for 7 d. **D** MDA concentrations in leaves of 15-d-old plants after 100 mM NaCl treatment for 24 h. **E** Relative ion leakage in leaves of 15-d-old plants after 100 mM NaCl treatment for 24 h. **F–H** Ion content in shoots of 4-week-old plants after 6 d of salt stress (100 mM NaCl). Wild type and *STK*-OE plants were exposed to salt stress (100 mM NaCl) for 6 d and the shoots were harvested for analysis of ion content. The Na^+^ and K.^+^ contents in seedlings were detected as previously described (Schmidt et al. 2013). Data in **B**–**H** are presented as mean ± SD (n = 3, **P ≤ 0.01, Tukey’s test)

**Fig. 4:**
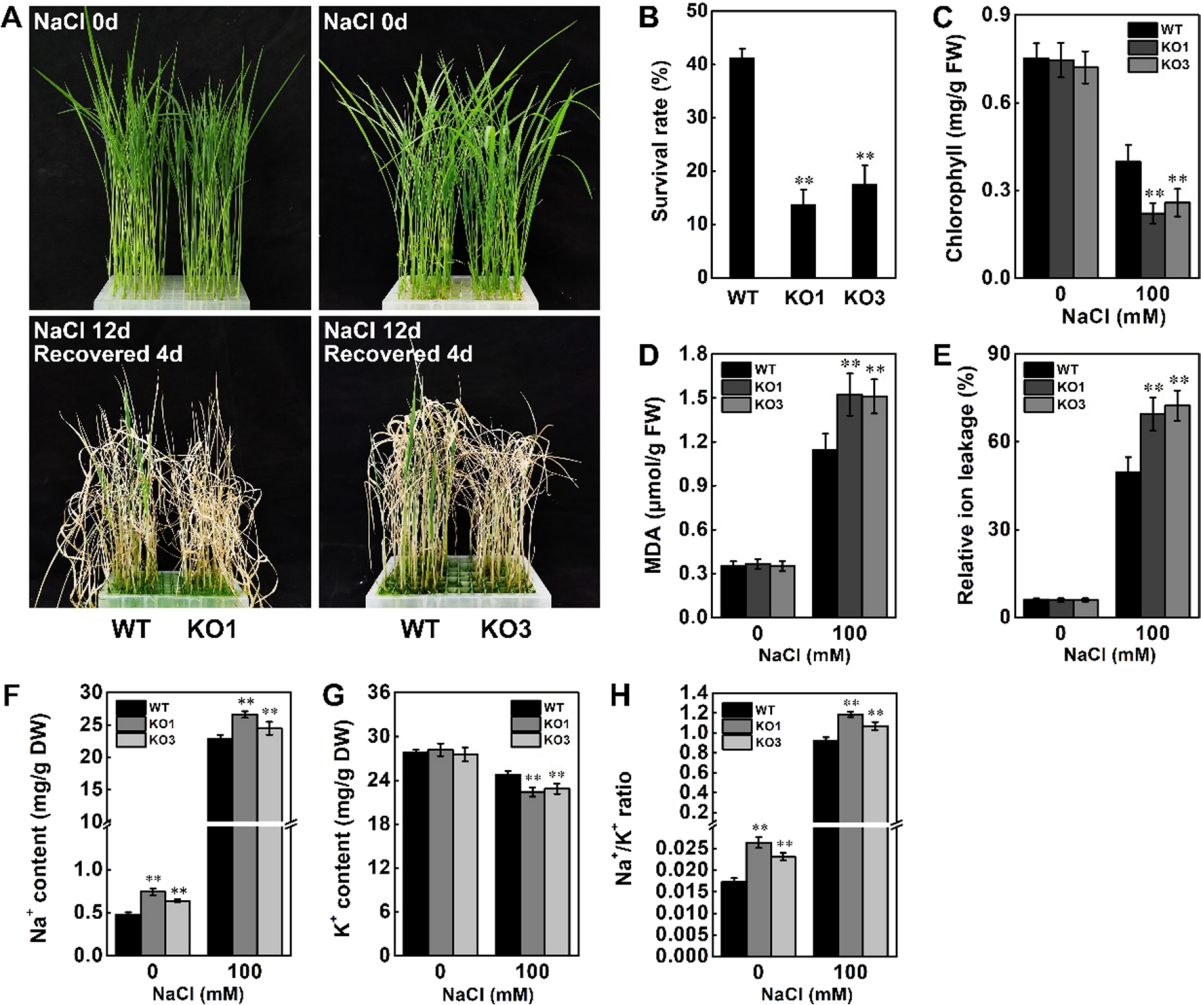
Knockout of *STK* increased salt sensitivity in rice. **A** Phenotype of WT, KO1 and KO3 rice plants under salt stress at seedling stage. For *STK*-KO plants, 15-d-old seedlings were treated with 140 mM NaCl for 12 d and recovery 4 d. **B** Survival rates of WT, KO1 and KO3 in (A) after 4 d of recovery. **C** Chlorophyll content in leaves of 15-d-old plants treated with 100 mM NaCl for 7 d. **D** MDA concentrations in leaves of 15-d-old plants after 100 mM NaCl treatment for 24 h. **E** Relative ion leakage in leaves of 15-d-old plants after 100 mM NaCl treatment for 24 h. **F**–**H** Ion content in shoots of 4-week-old plants after 6 d of salt stress (100 mM NaCl). Wild type and *STK*-KO plants were exposed to salt stress (100 mM NaCl) for 6 d and the shoots were harvested for analysis of ion content. The Na^+^ and K.^+^ contents in seedlings were detected as previously described (Schmidt et al. 2013). Data in (B) to (H) are presented as mean ± SD (n = 3, **P ≤ 0.01, Tukey’s test)

Under salt stress using 140 mM NaCl for 12 d and recovery 4 d, *STK*-OE lines showed greater resistance than wild-type plants (Fig. 3A). Almost 70% to 73% of the *STK*-OE plants survived, while only 43% of the wild-type plants survived under this treatment (Fig. 3B). As shown in Fig. 3C–E, compared with wild-type plants, *STK*-OE plants accumulated more chlorophyll and less malondialdehyde (MDA) and relative ion leakage. In contrast, when wild-type and *STK*-KO plants were subjected to 140 mM NaCl for 12 d, 41% of the wild-type plants recovered 4 d after watering was restored, but only 14% to 18% of the *STK*-KO plants recovered (Fig. 4A, B). Further physiological analyses revealed that loss of function of *STK* reduced the content of chlorophyll and increased levels of MDA and relative ion leakage in leaves (Fig. 4C–E). Previous studies showed that the ability to avoid Na^+^ excessive accumulation and keep a low Na^+^/K^+^ ratio contributes to salt tolerance in plants (Zhu 2003). We further measured Na^+^ and K^+^ contents in the shoot of wild-type, *STK*-OE and *STK*-KO plants. Less Na^+^ but higher K^+^ content was detected in *STK*-OE plants under salt stress, resulting in a lower Na^+^/K^+^ ratio in the shoot of *STK*-OE plants than wild-type (Fig. 3F–H). In contrast, compared with the wild-type plants, the *STK*-KO plants accumulated more Na^+^ and less K^+^, leading to a higher Na^+^/K^+^ ratio under salt stress (Fig. 4F–H). These findings indicated that *STK* confers tolerance to salt stress via relief of the membrane damage caused by salt stress and avoidance of Na^+^ excessive accumulation in plants.

### Overexpression of *STK* Enhances ABA Sensitivity

**Fig. 5:**
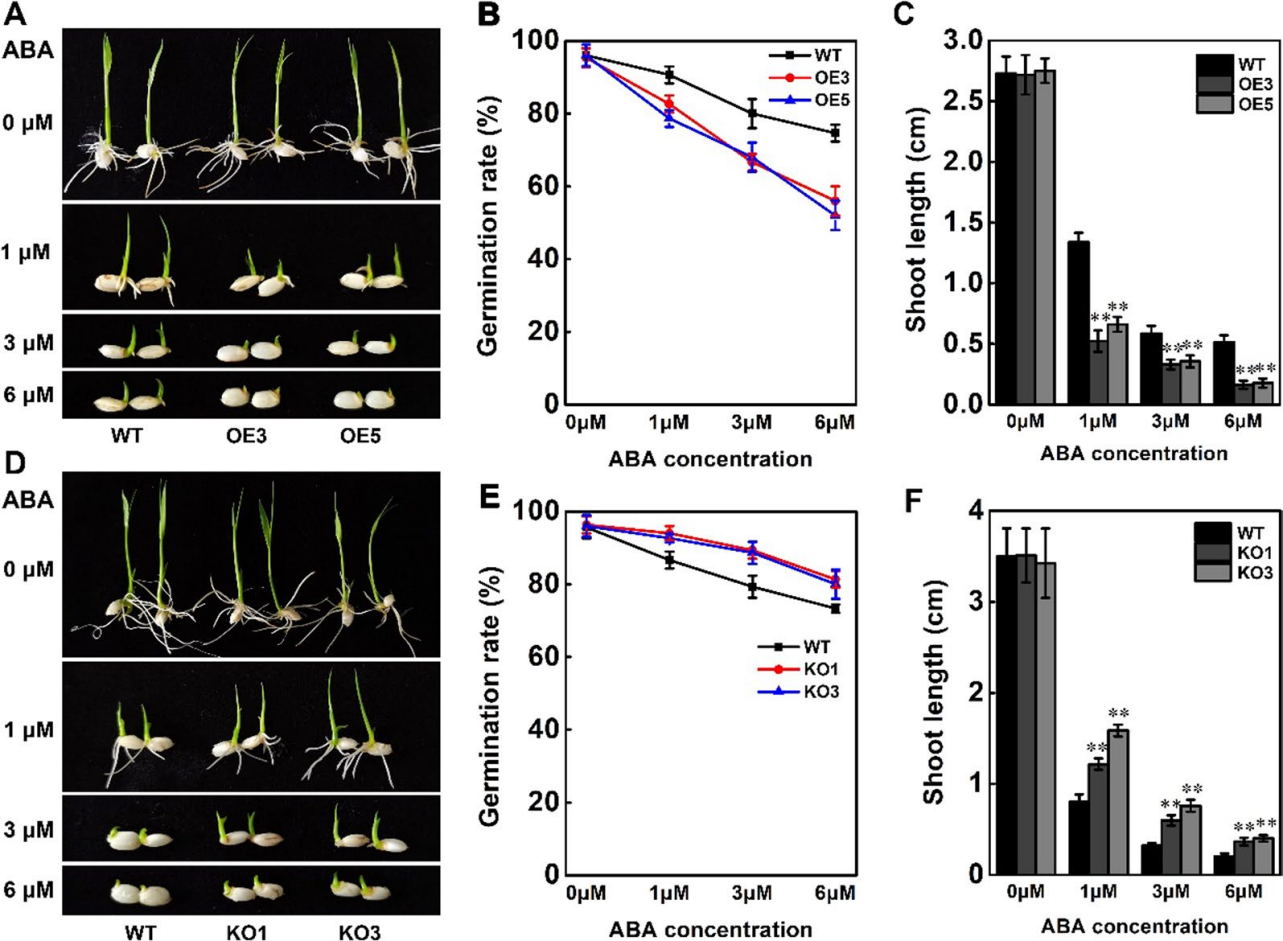
Increased ABA sensitivity of *STK* overexpression plants at germination stage. **A**, **D** Germination performance of *STK*-OE (**A**) and *STK*-KO (**D**) seeds on 1/2MS medium containing 0, 1, 3, or 6 μM ABA at 5 d after initiation. **B**, **E** The germination rates of *STK*-OE (**B**) and *STK*-KO (**E**) seeds after 0, 1, 3, or 6 μM ABA treatment. **C**, **F** Shoot lengths of *STK*-OE (**C**) and *STK*-KO (**F**) plants after 0, 1, 3, or 6 μM ABA treatment for 5 d. Data in (**B**, **C**, **E**, **F**) are presented as mean ± SD (n = 3, **P ≤ 0.01, Tukey’s test)

Recent study revealed that *STK* was up-regulated under ABA treatment (Gao and Xue 2012). As shown previously, the expression of *STK* was also induced by ABA (Fig. 1A). Consequently, we determined whether *STK*-OE plants and *STK*-KO plants showed differences in performance under ABA treatment compared with wild-type plants. The ABA sensitivity of rice plants was investigated at the germination stage. Seeds of *STK*-OE plants, *STK*-KO plants, and the wild-type were germinated in one-half-strength Murashige and Skoog (1/2MS) medium containing ABA with a gradient of concentrations (0, 1, 3 and 6 μM). The germination rate of the *STK*-OE plants and *STK*-KO plants was identical to the wild-type at 0 μM ABA treatment (Fig. 5A, B, D, E). However, compared with the wild-type, the germination rate was reduced in *STK*-OE plants and increased in *STK*-KO plants under 1, 3 and 6 μM ABA treatment, respectively (Fig. 5B, E). We also investigated the ABA sensitivity of rice plants at the post-germination stage. Compared with the wild-type, the shoot length was significantly shorter in *STK*-OE plants but markedly longer in *STK*-KO plants under 1, 3 and 6 μM ABA treatment, respectively (Fig. 5C, F). No significant difference in shoot length was observed among *STK*-OE, *STK*-KO and wild-type under without ABA treatment (Fig. 5C, F). These results suggested that STK is a positive regulator of ABA signaling in rice.

### STK Positively Regulates Oxidative Tolerance in Rice

**Fig. 6:**
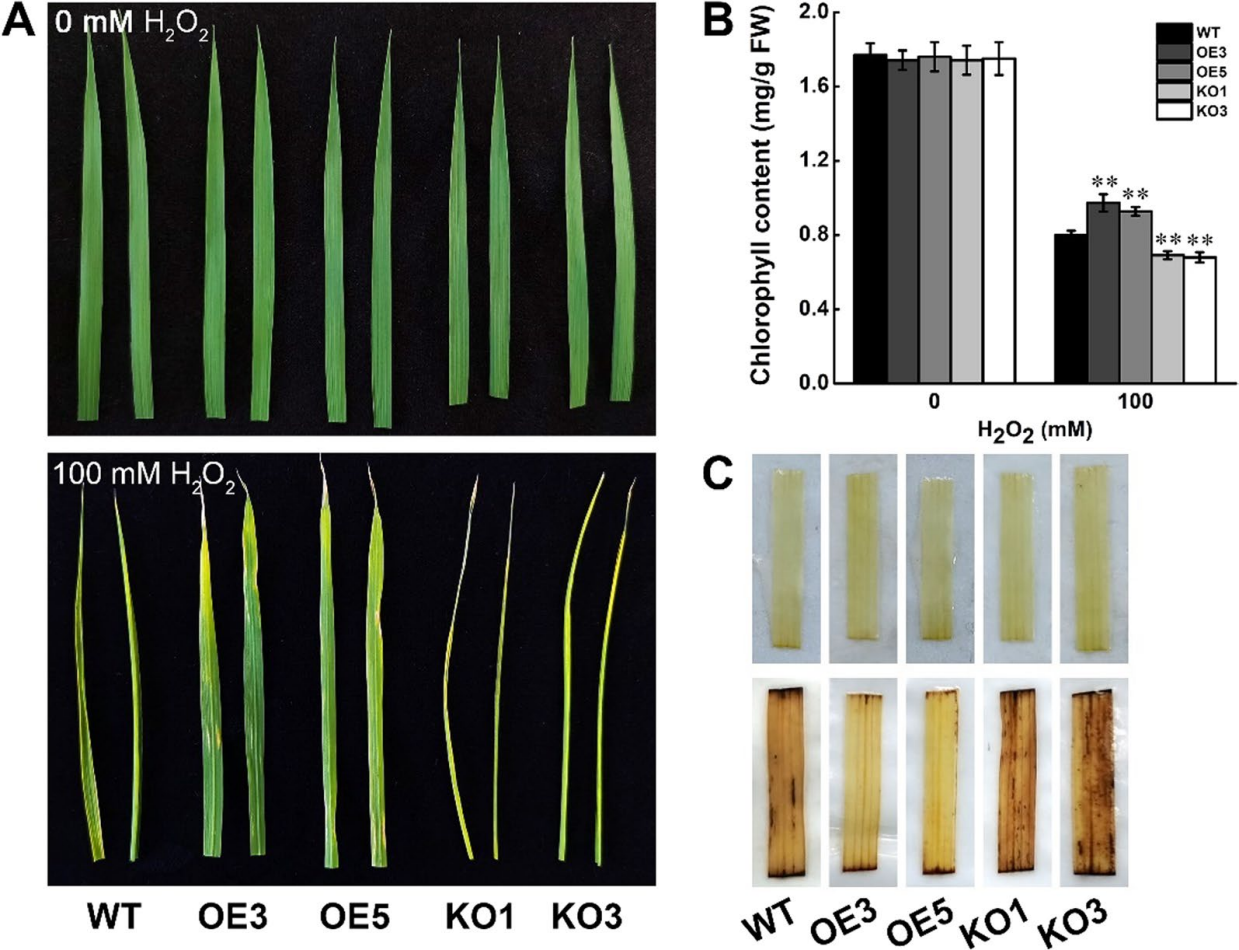
Increased oxidative tolerance of *STK* overexpression plants. **A** Leaf phenotype of WT*, STK-*OE and *STK-*KO plants at the three-leaf stage under normal conditions or after 100 mM H_2_O_2_ stress for 2 d. **B** Total chlorophyll contents of the WT*, STK-*OE and *STK-*KO plants under normal and H_2_O_2_ stress conditions. FW, Fresh weight. Data are presented as mean ± SD (n = 3, **P ≤ 0.01, Tukey’s test). **C** DAB staining for H_2_O_2_ in leaves from normal and H_2_O_2_-stressed WT*, STK-*OE *and STK-*KO plants for 1 d

Salt stress usually cause damage in plants via oxidative stress involving the generation of reactive oxygen species (ROS), such as H_2_O_2_ (Chen 2021). As shown previously, the expression of *STK* was induced by H_2_O_2_ (Fig. 1A) and *STK*-OE plants improved the salt tolerance of transgenic rice plants, we examined whether *STK* functions in salt tolerance through detoxification of ROS. Three-leaf stage seedlings of *STK*-OE plants, *STK*-KO plants and wild-type were treated with 100 mM H_2_O_2_. After 2 d H_2_O_2_ treatment, severe necrosis and leaf rolling were observed in *STK*-KO plants (Fig. 6A). Moreover, wild-type plants exhibited stronger leaf rolling and more chlorosis than *STK*-OE plants after H_2_O_2_ treatment. Then, the chlorophyll content in these seedlings were determined. Under oxidative stress, *STK*-OE plants showed higher chlorophyll content compared with wild-type, whereas the *STK*-KO plants exhibited the opposite results (Fig. 6B). Nevertheless, the chlorophyll content was not significantly different among *STK*-OE plants, *STK*-KO plants and wild-type under normal growth conditions. The accumulation of H_2_O_2_ was further determined by 3,3′-diaminobenzidine (DAB) staining. After 1d H_2_O_2_ treatment, the intensity of DAB staining was less in leaves of the *STK*-OE plants than in those of wild-type plants (Fig. 6C). The intensity of DAB staining was greater in the leaves of *STK*-KO plants than in those of wild-type plants. Thus, the *STK*-OE plants and the *STK*-KO plants showed opposite trends in ROS accumulation. Under normal growth conditions, no obvious staining was observed in *STK*-OE, *STK*-KO and wild-type. These results suggest that *STK* is involved in the elimination of H_2_O_2_ produced under oxidative stress.

### Identification of Stress-Related Genes Controlled by *STK*

**Fig. 7:**
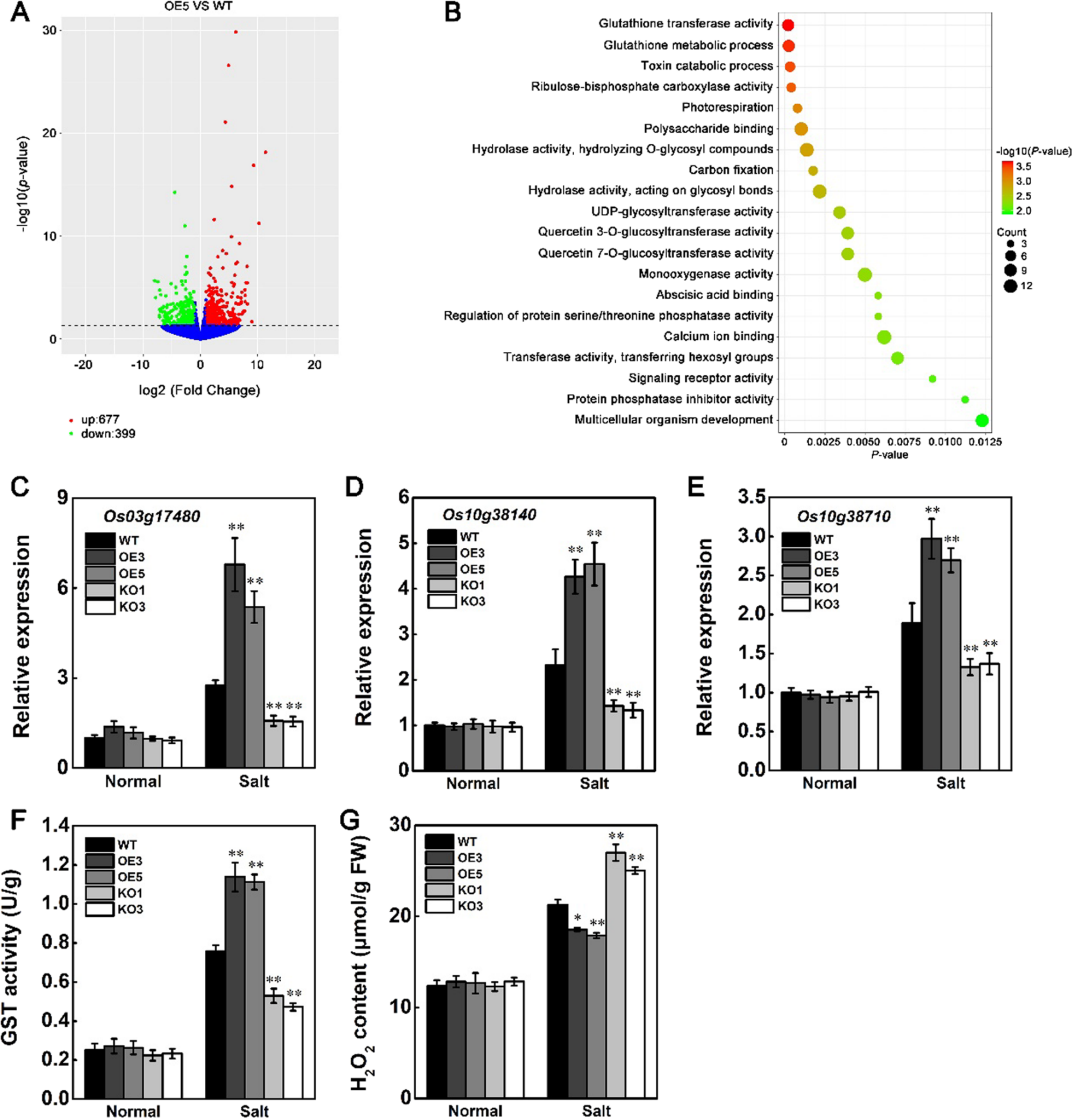
Differentially expressed genes (DEGs) in the *STK*-OE plants. **A** Volcano plots comparing the transcriptomes of OE5 plants with the WT. X-axis and Y-axis represent log_2_ fold change (FC) and − log_10_ (p-value), respectively. The green and red dots represent downregulated DEGs and upregulated DEGs, respectively. The blue dots represent no significant difference in transcriptomes. **B** Gene ontology (GO) enrichment analysis of DEGs. TOP 20 significantly enriched biological process GO terms are show. Three biological replicates were included for each treatment. **C**–**E** Relative expression of GST genes *Os03g17480* (**C**) *Os10g38140* (**D**) and *Os10g38710* (**E**) in rice leaves of seedlings grown under normal conditions or after 150 mM NaCl for 48 h. **F** GST activity in rice leaves of seedlings grown under normal conditions or after 150 mM NaCl for 48 h. **G** H_2_O_2_ contents in rice leaves of seedlings grown under normal conditions or after 150 mM NaCl for 48 h. Data in (**C**–**G**) are presented as mean ± SD (n = 3, *P ≤ 0.05, **P ≤ 0.01, Tukey’s test)

To understand the mechanisms and downstream components of the *STK*-mediated salt-stress signaling pathways, transcriptome deep sequencing (RNA-seq) analysis was performed using leaves from wild-type and *STK*-OE (OE5) rice plants under salt stress conditions. The threshold for significantly differentially expressed genes (DEGs) was set at a (log2 scale)-fold change (FC) value of > 1 or <  − 1 and adjusted *p*-value < 0.05. Using these criteria, we identified 1076 DEGs (677 upregulated and 399 downregulated) in OE5 compared to wild-type. These DEGs altered by salt stress are shown in the volcano plots, which illustrate the asymmetry between upregulated (red) and downregulated (green) DEGs (Fig. 7A). Gene ontology (GO) enrichment analysis showed that the DEGs affected by the overexpression of *STK* were enriched mainly in glutathione transferase activity and glutathione metabolic process (Fig. 7B). The GST (Glutathione S-transferase) genes (*LOC_Os03g17480*, *LOC_Os10g38140* and *LOC_Os10g38710*) detected in the RNA-seq were further investigated for their expression changes in the *STK*-OE, *STK*-KO and wild-type under normal and salt stress conditions by RT-qPCR. Under normal conditions, no significant differences in expression levels were observed among the *STK*-OE, *STK*-KO and wild-type. On the contrary, compared with the wild-type plants, the expression levels of the GST genes were significantly increased in the *STK*-OE plants but markedly lower in *STK*-KO plants under salt stress conditions (Fig. 7C–E). GST is the ubiquitous enzymes that play a key role in cellular detoxification (You et al. 2014). Thus, *STK* maybe involved in the detoxification and ROS scavenging mechanisms of rice by GST under salt stress. To check this hypothesis, GST activities and ROS levels in the salt-stressed and unstressed leaves of *STK*-OE, *STK*-KO and wild-type were measured. Compared with the wild-type, GST activity was significantly higher in the *STK*-OE plants but markedly lower in *STK*-KO plants under salt stress conditions (Fig. 7F). Similarly, the H_2_O_2_ content was also significantly lower in *STK*-OE plants but markedly higher in *STK*-KO plants under salt stress conditions (Fig. 7G). Under normal growth conditions, no significant difference of GST activity and H_2_O_2_ content were detected among the *STK*-OE, *STK*-KO and wild-type plants, respectively. These results indicated that *STK* was involved in the detoxification and ROS scavenging mechanisms of rice under salt stress conditions, which may have contributed to their improved tolerance to salt stress.

Previously identified several stress-responsive genes involved in various pathways, including *OsABAR1*, *Os3BGlu6*, *OSBZ8* and *OsSIK1* (Mukherjee et al. 2006; Ouyang et al. 2010; Wang et al. 2020a, b; Zheng et al. 2020) were up-regulated at 48 h of salt stress treatment (Additional file 2: Table S2). Expression levels of these genes under normal or salt conditions was further verified by RT-qPCR in *STK*-OE, *STK*-KO and wild-type plants. In agreement with the analysis of DEGs, the expression of these genes was up-regulated and downregulated in *STK*-OE plants and *STK*-KO plants under salt stress condition, respectively (Additional file 1: Fig. S3). These results suggested that *STK* may promote salt tolerance through activated stress-related genes.

## Discussion

Excessive accumulation of ROS is a frequent event in plants suffering from diverse abiotic stresses, including drought, high salinity, and extreme temperatures, and can cause damage to plants (Mittler 2002; Miller et al. 2010). Overaccumulation of ROS can damage DNA, proteins and carbohydrates, resulting in cell death (Mittler et al. 2004). ROS also cause MDA production, cell membrane damage and lipid peroxidation. Previous studies showed that the plant response to abiotic stresses occurs through the regulation of ROS metabolism (Schmidt et al. 2013; Li et al. 2014; Fang et al. 2015; Zhou et al. 2018; Zhao et al. 2019). For example, Salt Intolerance 1 (SIT1), a rice L-type LecRLK, could be activated by salt and mediate salt sensitivity by activation of MPK3/MPK6 leading to higher ethylene production and downstream ROS accumulation (Li et al. 2014; Zhao et al. 2019). Overexpression of a NAC protein, SNAC3, improves heat and drought tolerance by modulating ROS homeostasis through regulation of the expression of genes encoding ROS production and ROS-scavenging enzymes (Fang et al. 2015). STRK1 (Salt Tolerance Receptor-like Cytoplasmic Kinase 1), which activates CatC activity through phosphorylation at Tyr-210 of CatC to regulate H_2_O_2_ homeostasis and improve salt tolerance (Zhou et al. 2018). In this study, induction of *STK* upon H_2_O_2_ treatment indicated that it functions in oxidative stress adaptation (Fig. 1A). Reduced MDA levels in the *STK*-OE plants under salt stress treatment (Fig. 3D) implied that less oxidative damage occurred in the cells of the *STK*-OE plants during the salt stress conditions. Relevant to this finding, *STK*-OE plants showed better growth under oxidative stress caused by H_2_O_2_ (Fig. 6A, B). In *STK*-OE plants, one ROS, H_2_O_2_, was present in low levels under salt stress, as revealed by DAB staining (Fig. 6C). To the contrary, *STK*-KO plants showed reduced tolerance to oxidative stress. These results suggested that the function of STK in salt tolerance may be associated with the regulation of antioxidation ability.

To maintain cellular redox homeostasis and scavenge excess stress-induced ROS, plants have evolved antioxidation systems comprising nonenzymatic and enzymatic antioxidants (Miller et al. 2010). The maintenance of high activity of various antioxidant enzymes, such as SOD, CAT, GST, GR, and glutathione peroxidase, to scavenge the toxic ROS has been linked to improved tolerance of plants to abiotic stresses (Apel and Hirt 2004; Mittler 2002; Miller et al. 2010). GST plays an important role in the reduction of organic hydroperoxides formed during oxidative stress using the tripeptide glutathione (GSH) as a cosubstrate or coenzyme (Dixon et al. 2002). Overexpression of a GST gene from *Escherichia coli* in transgenic tobacco enhanced salt and cold tolerance (Le Martret et al. 2011). The *ospp18* mutant showed increased drought and oxidative sensitivity through reduced the expression of GST genes and the activity of GST which significantly contribute to excessive H_2_O_2_ accumulation (You et al. 2014). Overexpression of a GST gene (*ThGSTZ1*) from *Tamarix hispida* improves drought and salinity tolerance by enhancing the ability to scavenge ROS (Yang et al. 2014). In the present study, the RNA-seq assay revealed that under salt conditions, *STK* increased the expression of GST genes (*LOC_Os03g17480*, *LOC_Os10g38140* and *LOC_Os10g38710*) and further confirmed by RT-qPCR. Under salt stress, the expression of GST genes and the activity of GST were found to be higher in the *STK*-OE plants than in wild-type, and *STK*-KO plants showed the reverse results (Fig. 7C–F). The low level of H_2_O_2_ in *STK*-OE plants was most likely a result of high levels of the enzyme GST (Fig. 7G). These results suggested that the enhanced activity of ROS scavenging enzymes may significantly contribute to scavenge H_2_O_2_ accumulation and reduce oxidative damage in *STK*-OE plants, which is associated with the increased tolerance of the plants to salt stress.

High concentrations of salt in plant cells can induce ionic stress. Ionic stress is caused by sodium (Na^+^) and chlorine (Cl^−^) accumulation in plant cells, eventually resulting in premature leaf senescence and even plant death (Liu et al. 2021). The toxicity of Na^+^ mainly manifested in its inhibitory effect on enzyme activities, negatively affecting metabolism, including the Calvin cycle and other pathways (Cheeseman 2013; Wu et al. 2018). Potassium (K^+^), a vital ion for plant growth, plays a critical role in salt tolerance regulation as the cytosolic Na^+^/K^+^ ratio appears to determine plant salt tolerance (Shabala and Cuin 2008; Wu et al. 2018). Na^+^ and K^+^ are imported into the plant cell using the same set of transporters, and the two cations compete with each other (Greenway and Munns 1980). In glycophytes, excessive Na^+^ commonly results in K^+^ deficiency under salt stress (Yang and Guo 2018). Therefore, keeping a low Na^+^/K^+^ ratio is an important mechanism used by plants to adapt to salt stress. OsSOS1, a rice plasma membrane Na^+^/H^+^ antiporter, excludes Na^+^ from the shoot, promoting a lower cellular Na^+^/K^+^ ratio and improving salt tolerance (Martínez-Atienza et al. 2007; El Mahi et al. 2019). The K^+^ channel OsAKT1 and OsKAT1 are involved in salt tolerance via maintaining a low Na^+^/K^+^ ratio owing to the increase of K^+^ uptake (Fuchs et al. 2005; Obata et al. 2007). Salt Intolerance 2 (SIT2), a rice LecRLK, was identified for its role in salinity stress tolerance potentially via its function in Na^+^ extrusion by regulating SOS pathway (Sun et al. 2020). Our results demonstrate that less Na^+^ but higher K^+^ content was detected in *STK*-OE plants under salt stress, resulting in a lower Na^+^/K^+^ ratio in the shoot of *STK*-OE plants than wild-type (Figs. 3H, 4H). These results indicated that STK may be involved in the transport of Na^+^ and K^+^, regulating rice tolerance to salt stress by avoiding Na^+^ accumulation and promoting K^+^ uptake.

A better understanding of the mechanisms underlying salt stress sensing and signal transduction will help researchers design novel approaches to increase plant tolerance to salt stress. In response to high salinity, the initial signal activates the production of compounds that trigger activity in many metabolic and developmental pathways (Li et al. 2019). One of the most important compounds is abscisic acid (ABA; Finkelstein and Gibson 2002). ABA plays an important role in regulating the response of plants to environmental stress (Sah et al. 2016; Vishwakarma et al. 2017). Salt stress can trigger the ABA-dependent signaling pathway in plants (Zhu 2002). *GmWRKY16* as a WRKY transcription factor may promote tolerance to drought and salt stresses through an ABA-mediated pathway (Ma et al. 2019). Overexpression of *OsSRK1* enhances salt tolerance probably by regulating stress-related genes and ABA pathway (Zhou et al. 2020). *OsNAC45* positively regulates ABA signal pathway and is required for salt tolerance in rice (Zhang et al. 2020). Overexpression of *PpSnRK1α* in tomato is beneficial for enhancing salt tolerance by regulating ABA signaling pathway and reactive oxygen metabolism (Wang et al. 2020a, b). ABA plays important roles in many aspects of plant development and physiological processes, such as seed maturation, germination, seedling growth and stomatal movement, is the central regulator of abiotic stress resistance in plants (Finkelstein et al. 2008; Kim et al. 2010). In the present study, an expression pattern analysis showed that *STK* expression was strongly induced by ABA (Fig. 1A), indicating that *STK* is involved in ABA signaling pathway. In the absence of ABA, the germination rates of the WT, *STK*-OE and *STK*-KO plants were similar, and no significant difference in shoot length was observed among wild-type, *STK*-OE and *STK*-KO plants after germinating (Fig. 5). In the presence of ABA, the germination and growth of the *STK*-OE plants were severely inhibited compared with the wild-type. By contrast, the *STK*-KO plants germinated more quickly and grew faster than the WT. This phenomenon may be in part explained by the RNA-seq data. *OsSAPK10* is a positive regulator of the ABA signal pathway in seed germination and early seedling growth (Wang et al. 2020a, b). *OsSAPK10* overexpression lines displayed more severe ABA-mediated repression on seed germination and seedling growth (Wang et al. 2020a, b). The RNA-seq showed that the expression of *OsSAPK10* in *STK*-OE plants is significantly higher than wild-type plants under salt stress conditions (Additional file 2: Table S2), which is further confirmed by RT-qPCR (Additional file 1: Fig. S4). These results showed that *STK* confers tolerance to salt stress through an ABA-dependent signaling pathway.

To illustrate the molecular mechanism underlying the enhanced salt tolerance of *STK*-OE plants, RNA-seq was performed. Under salt conditions, 677 genes were upregulated and 399 genes were downregulated (Fig. 7A). GO annotation analysis showed that these genes mainly belong to following categories: glutathione transferase activity and glutathione metabolic process (Fig. 7B). Many abiotic stress-related genes were regulated in *STK*-OE transgenic plants compared with WT (Additional file 2: Table S2). After salt treatment, the relative expression levels of *OsABAR1*, *Os3BGlu6*, *OSBZ8* and *OsSIK1* increased more obviously in *STK*-OE plants compared with wild-type. The *OsABAR1*-overexpressing plants showed increased tolerance to drought and salinity, whereas the *OsABAR1* knockout plants had the opposite phenotypes (Zheng et al. 2020). Overexpression of *OsSIK1* increased tolerance of rice plants to salt and drought stresses, and the knockout mutants *sik1-1* and *sik1-2*, as well as RNA interference (RNAi) plants, are sensitive to drought and salt stresses (Ouyang et al. 2010). Os3BGlu6, a chloroplast localized β-glucosidase, significantly affected cellular ABA pools, and playing an important role in drought stress and photosynthesis under normal growth and drought conditions (Wang et al. 2020a, b). OSBZ8, a bZIP class of ABRE-binding transcription factor, is shown to be highly expressive in response to tolerance against salt stress cultivars as compared to those that are sensitive to salt stress (Mukherjee et al. 2006). It is worth noting that previous reports have shown that OSBZ8 is a marker for ABA-responsive genes (Miyoshi et al. 1999; Nakagawa et al. 1996). Therefore, the higher expression levels of these stress-related genes in *STK*-OE transgenic plants could contributed to increased tolerance of the transgenic plants to salt stress.

## Conclusions

A novel rice RLCK gene, *STK*, which was rapidly induced by ABA as a prominent regulator of the response to salt stress while causing rice to be sensitive to exogenous ABA. Overexpression of *STK* improved tolerance to salt and oxidative stresses than wild-type. On the contrary, the knockout mutants *STK*-KO were sensitive to salt and oxidative stresses. The activity of GST was enhanced significantly in *STK*-OE plants. Also, the accumulation of H_2_O_2_ in leaves of *STK*-OE plants was much less than that of the *STK*-KO plants and wild-type. *STK* improved salt tolerance by inducing antioxidant defense and associated with the ABA signaling pathway in rice. In summary, *STK* enhanced ROS scavenging capacity by regulating the expression of GST genes, thereby increasing the salt tolerance of rice.

## Methods

### Plant Materials and Growth Conditions

Rice cultivars Kitaake (*Oryza sativa* ssp. *japonica*) were used for RT-qPCR analysis under various stresses and phytohormone treatments, and Kitaake was used for all transgenic experiments. For gene expression analysis, rice seeds were first sterilized with 15% NaClO, germinated at 30 °C for 3 days, and then grown in hydroponic culture solution (Ren et al. 2005) with a 14-h light/10-h dark photoperiod, a 28 °C (light)/25 ℃ (dark) temperature range, 350 μmol m^−2^ s^−2^ light intensity, and 85% relative humidity. Rice seedlings at the three-leaf stage were immersed with their roots in NaCl (150 mM), PEG (20%), H_2_O_2_ (1%), cold (4 °C), ABA (100 μM), GA (100 μM) for the indicated times. Their leaves were harvested at 0, 1, 3, 6, 12, and 24 h after the beginning of treatments. All harvested leaf samples were rapidly frozen in liquid nitrogen and stored at − 80 °C for RNA extraction.

### RNA Extraction and RT-qPCR Analysis

Total RNA was extracted from rice leaves using TRIzol reagent (Sangon Biotech, Shanghai, China) and treated with gDNA Eraser to eliminate any DNA contamination. The quality of the total RNA was evaluated using a NanoDrop 2000 (Thermo Fisher Scientific, Shanghai, China). First-stand cDNAs were synthesized using PrimeScript RT reagent kit (Takara, Beijing, China) following the manufacturer’s instruction. RT-qPCR was performed with ABI 7500 Real Time PCR System (Applied Biosystems, Foster City, USA) using TB Green Premix Ex Taq II (Takara, Beijing, China) to monitor dsDNA synthesis, according to the manufacturer’ s instruction. The primers used for RT-qPCR are listed in Additional file 2: Table S1. The expression of rice Actin gene (LOC_Os03g50885) was used as an internal control. The relative expression levels were measured as previously described (Zhou et al. 2022).

### Plasmid Constructions and Generation of *STK*-OE and *STK*-KO Plants

To generate the *STK*-OE plants, the coding region of the *STK* was cloned in the *BamH*I and *EcoR*I sites of the pCAMBIA1300-YFP vector using a specific primer (Additional file 2: Table S1). To generate the *STK*-KO plants, the target sites of *STK* were selected by the CRISPR-Plant Web server (Xie et al. 2014) and were constructed into the CRISPR/Cas9 vector as described previously (Zhou et al. 2022). The plasmids were introduced into *Agrobacterium tumefaciens* strain EHA105. *Agrobacterium*-mediated transformation of embryogenic calli generated from the *japonica* rice cultivar Kitaake was performed as previously described (Zhou et al. 2015). To determine whether genome editing of *STK* was successful, genomic DNA extracted from regenerated transgenic plants was amplified by PCR with the primers listed in Additional file 2: Table S1, followed by sequencing of the PCR products.

### Stress Treatments

For salt stress tolerance assays, uniformly germinated seeds of WT, *STK*-OE, and *STK*-KO lines were grown in hydroponic culture solution. 15-d-old plants (40 plants each genotype) were transferred to a hydroponic culture solution containing 140 mM NaCl, and 12 d NaCl treatments were applied for *STK*-KO and *STK*-OE plants as well as their corresponding wild-type, respectively. The hydroponic solution was changed every two days during salt stress treatment. Then, the plants were transferred to a normal hydroponic culture solution to recover for 4 d, and the color of the leaves was observed. If the leaves were green, they were counted as surviving plants, if the leaves were withered brown, they were counted as dead plants, and finally the survival rate was counted.

For testing the ABA sensitivity of transgenic plants at the germination stage, seeds of WT, *STK*-OE, and *STK*-KO lines (30 seeds each, three repeats) were sterilized with 15% NaClO and cultured on hydroponic culture solution containing a gradient concentration of ABA (0, 1, 3, and 6 μM). After 5 d treatment, the number of germinated seeds with emerged coleoptiles and germination rate were calculated.

For the H_2_O_2_ treatment, three-week-old plants (30 plants each genotype) were irrigated with hydroponic culture solution containing 100 mM H_2_O_2_ solution. After 1 d, the leaves were stained with DAB following the previously described method (You et al. 2014). After 2 d, the leaves were photographed.

### Physiological Measurements

The total chlorophyll content was determined according to the method described previously (Zhou et al. 2018). About 100 mg of leaf blades were ground in liquid nitrogen and then immersed in the extract solution (80% acetone) under darkness overnight at 4 °C. The absorbance of the extracts was read at 663 and 645 nm with a spectrophotometer (BioTek). The total chlorophyll content was calculated and expressed as mg g^−1^ FW. The content of MDA was measured as previously described (Ouyang et al. 2010) with slight modification. About 100 mg of leaf blades were homogenized in 1 mL of 10% trichloroacetic acid (TCA). The homogenate was centrifuged at 5000 g for 10 min at 4 °C. To 800 μl of the supernatant was added 800 μl of thiobarbituric acid (TBA) (made in 10% TCA). The mixture was boiled for 15 min and centrifuged at 12,000*g* at 4 °C for 5 min. The absorbance of the supernatant was read at 450, 532, and 600 nm. The MDA content was determined using the extinction coefficient of 155 (nmol/L/cm) and expressed as mmol g^−1^ FW. The relative ion leakage was assayed according to the method described previously (Cao et al. 2007). The activity of glutathione S-transferase (GST) and the concentration of H_2_O_2_ were measured as described by using rice leaf blades using commercial kits (D799774-0100, Sangon Biotech, Shanghai, China; D799612-0100, Sangon Biotech, Shanghai, China) according to the manufacturer’s instructions. One unit of GST activity was defined as the amount of enzyme depleting 1 μM GSH in 1 min. H_2_O_2_ was detected by DAB staining as described previously (Zhou et al. 2018).

### Subcellular Localization of STK

For detection of the subcellular localization of STK, the coding sequences of the *STK* were fused to yellow fluorescent protein (YFP) reporter coding sequences and subcloned into the pCAMBIA1300-YFP vector, in which the YFP-coding sequence was fused in frame to the 3′ end of the *STK* gene sequence. The plasmid was transformed into isolated rice protoplasts using polyethylene glycol (PEG)-mediated transformation methods (Zhou et al. 2018).

Protoplasts were then transferred into multi-well plates and cultured in the dark at room temperature for 6–16 h. After incubation, green fluorescence signals from transfected protoplasts were observed using a confocal laser scanning microscope (Olympus FV1000) with 488 nm excitation and 505–530 nm emission wavelengths.

### RNA Sequencing (RNA-seq) Analysis

For RNA-seq, three-week-old seedlings of WT and *STK*-OE plants were treated with 150 mM NaCl for 48 h. Total RNA was extracted from leaves of three-week-old WT and *STK*-OE plants with the TRIzol reagent (Sangon Biotech, Shanghai, China) according to the manufacturer’s instructions. Library preparation and Illumina sequencing was performed at Novogene from three biological repeats of samples. Raw reads were trimmed by cutadapt to get clean reads. The trimmed reads were then mapped onto the reference rice genome from RAP-DB (https://rapdb.dna.affrc.go.jp). DEGs responding to salt stress were defined by a ≥ twofold expression change and p-value < 0.05, and genes which were up regulated between WT and OE plants were subjected to further gene ontology (GO) enrichment analysis using the ClusterProfiler (Yu 2018). The expression levels of selected DEGs were verified by RT-qPCR.

## Supplementary Information

**Additional file 1. Fig. S1:** Identification of CRISPR/Cas9-STK rice mutant plants. **Fig. S2:** Characterization of STK overexpressionplants. **Fig. S3:** Relative expression levels of four previously known stress-related genes up-regulated in STK-OE pants and down-regulated in STK-KO plants at 48 h under salt stress condition in rice seedlings. **Fig. S4:** Relative expression levels of OsSAPK10 in leaves of wild-type, STK-OE and STK-KO plants under salt stress condition.

**Additional file 2. Table S1:** List of primers used in this study. **Table S2:** Differential expression data of stress-related in the STK-OE plants.

## Data Availability

All data supporting the findings of this study are available from the corresponding author on reasonable request.

## Abbreviations

RLK: Receptor-like kinase
RLCK: Receptor-like cytoplasmic kinase
OE: Overexpression
KO: Knockout
WT: Wild-type
YFP: Yellow fluorescent protein
ABA: Abscisic acid
ROS: Reactive oxygen species
RT-qPCR: Reverse transcription quantitative PCR
DAB: 3,3′-Diaminobenzidine
GST: Glutathione S-transferase
GO: Gene ontology
DEG: Differentially expressed gene
FDR: False discovery rate
SD: Standard deviation
