# Supplementary material for: Receptor-Like Cytoplasmic Kinase STK Confers Salt Tolerance in Rice
Source: Rice (N Y). 2023 Apr 21;16:21. doi: 10.1186/s12284-023-00637-0 (PMC10121980; doi:10.1186/s12284-023-00637-0)
Supplement: Supplementary file 1 — Additional file 1. Fig. S1: Identification of CRISPR/Cas9-STK rice mutant plants. Fig. S2: Characterization of STK overexpressionplants. Fig. S3: Relative expression levels of four previously known stress-related genes up-regulated in STK-OE pants and down-regulated in STK-KO plants at 48 h under salt stress condition in rice seedlings. Fig. S4: Relative expression levels of OsSAPK10 in leaves of wild-type, STK-OE and STK-KO plants under salt stress condition. [file 12284_2023_637_MOESM1_ESM.docx]

**Additional information**


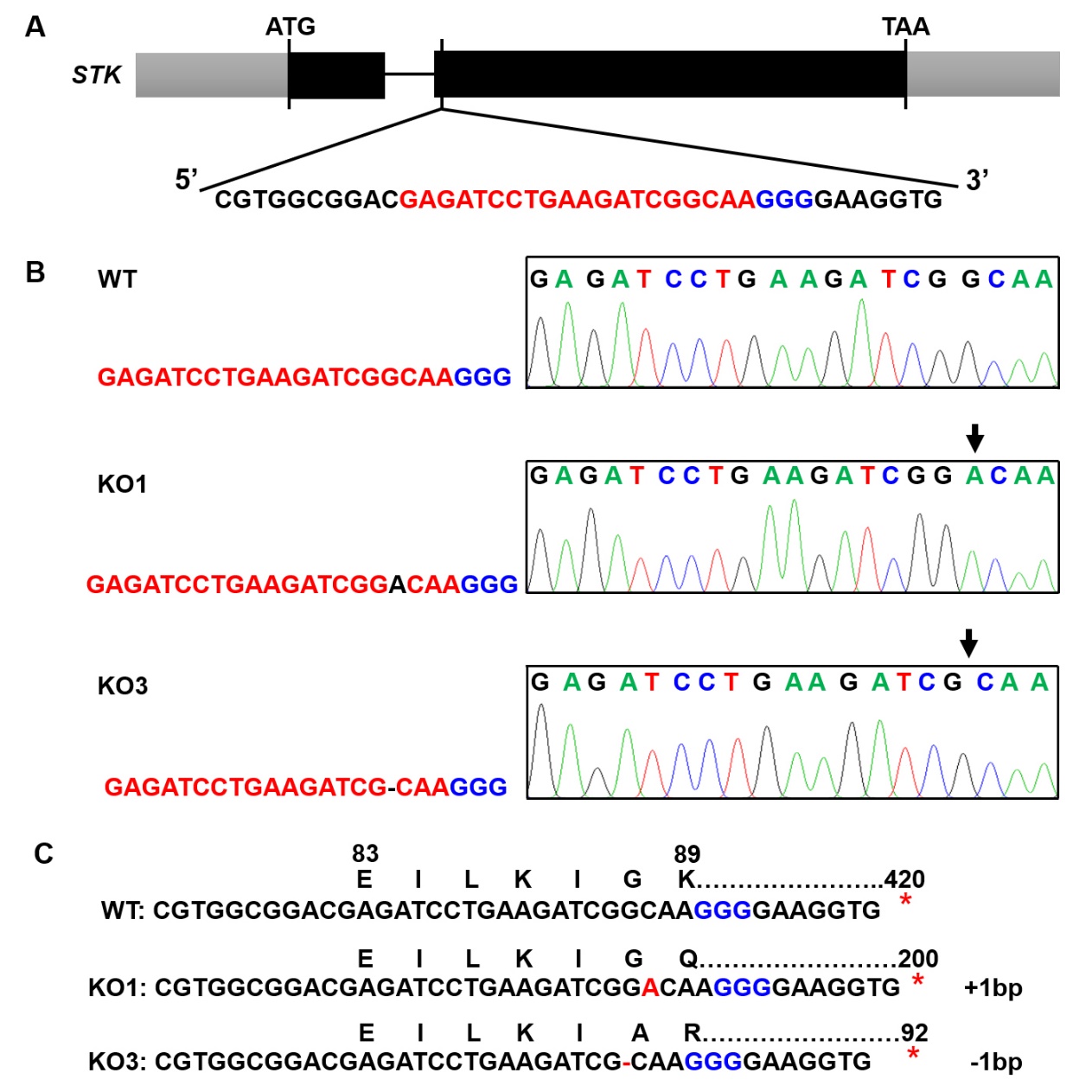


**Fig. S1** Identification of CRISPR/Cas9-*STK* rice mutant plants. **A:** Schematic of the *STK* gene structure and target site. Exons and introns are indicated with black rectangles and black lines, respectively. The spacer and PAM sequences were marked in red and blue. **B:** Homozygous mutations identified at the target site of *STK*-KO plants in the T_1_ generation. **C:** Amino acids of STK in *STK*-KO plants. Amino acids were marked above the relative nucleotide triplets, and the first altered ones from the frameshift were indicated in red, with the number representing the order in the proteins. The stars represent premature stop codons.


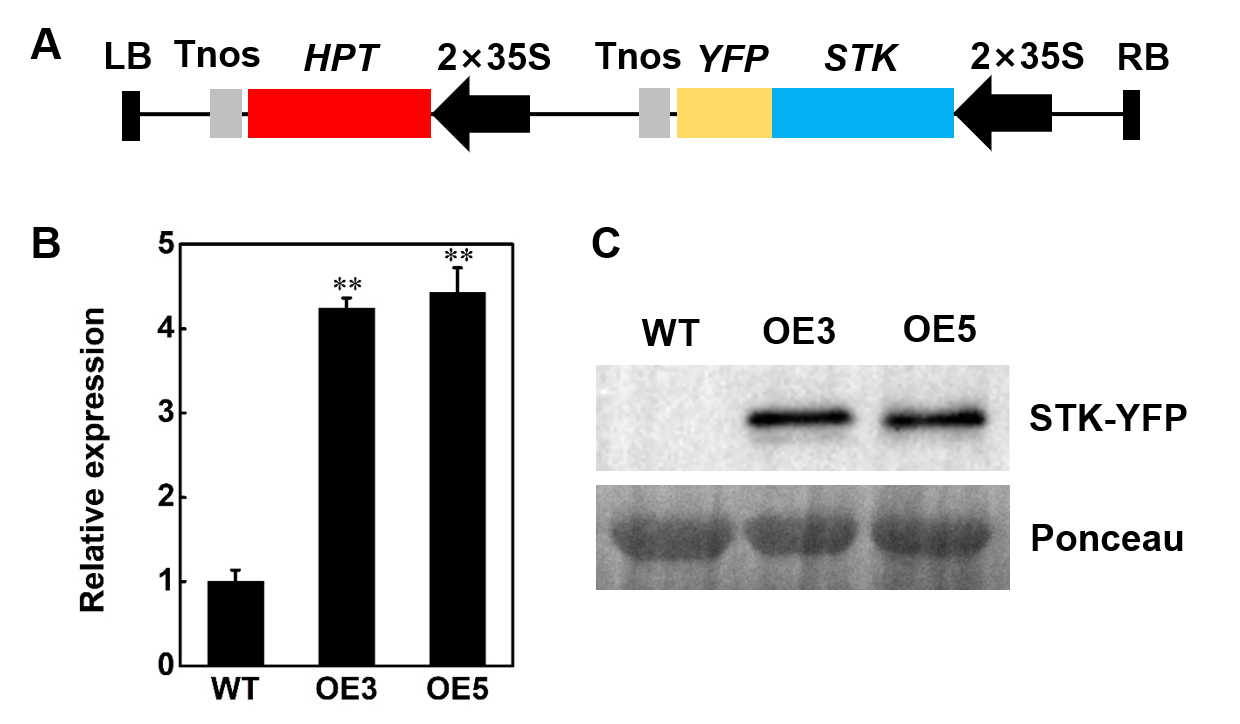


**Fig. S2** Characterization of *STK* overexpression (*STK*-OE) plants. **A:** Schematic representation of the overexpression constructs used for *STK*-OE assay. **B:** RT-qPCR analysis of *STK* transcript levels in wild-type, OE3 and OE5 plants. The *ACTIN* gene was amplified as a control. Data are presented as mean ± SD (n = 3, ***P* ≤ 0.01, Tukey’s test). **C:** Western blot analysis of the STK-YFP protein in wild-type (WT), OE3 and OE5 plants using anti-GFP antibody.


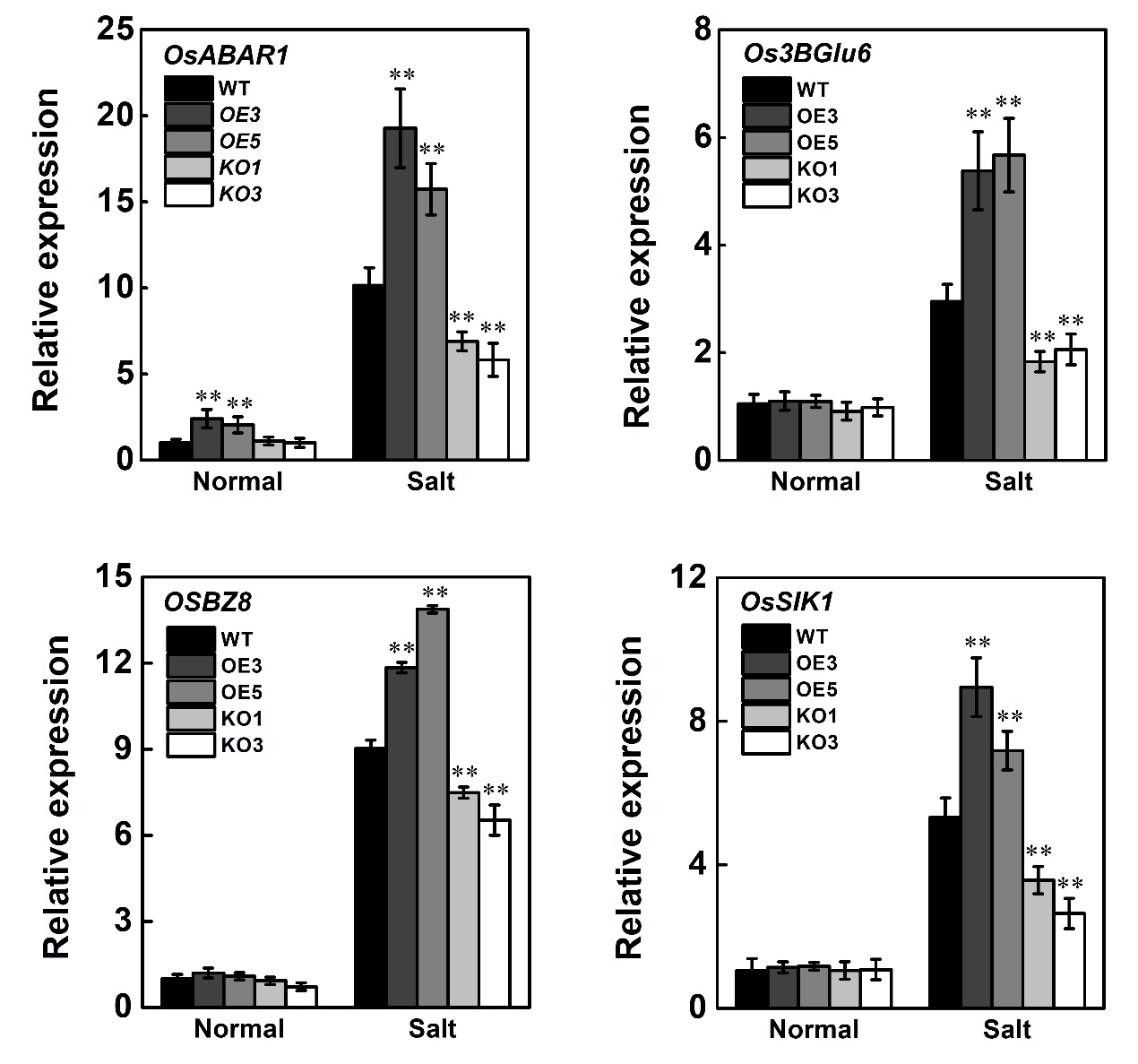


**Fig. S3** Relative expression levels of four previously known stress-related genes up-regulated in *STK*-OE pants and down-regulated in *STK*-KO plants at 48 h under salt stress condition in rice seedlings. Values are means ± SD (n = 3, ***P* ≤ 0.01, Tukey’s test).


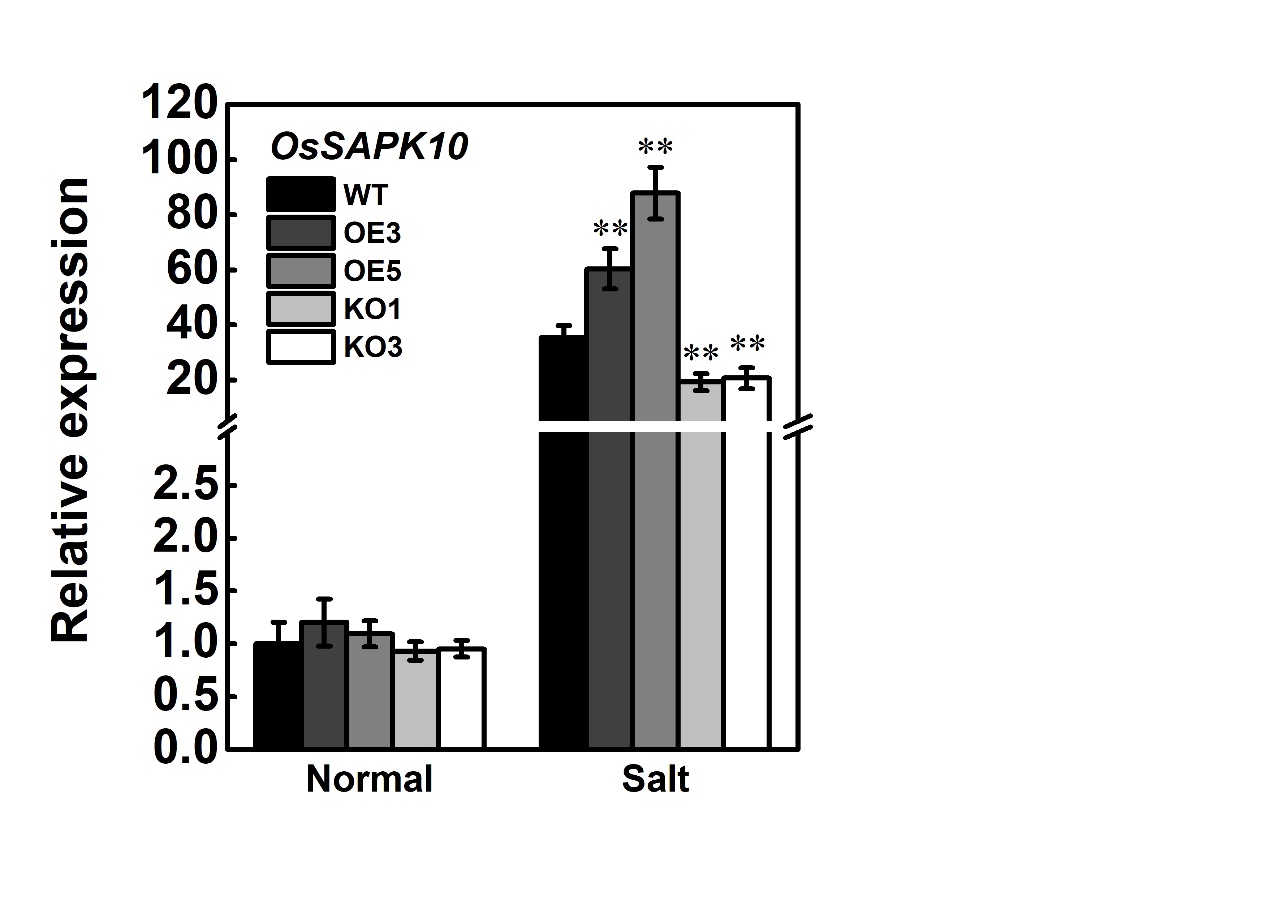


**Fig. S4** Relative expression levels of *OsSAPK10* in leaves of wild-type, *STK*-OE and *STK*-KO plants under salt stress condition. Values are means ± SD (n = 3, ***P* ≤ 0.01, Tukey’s test).
