# Supplementary material for: Receptor-Like Cytoplasmic Kinase STK Confers Salt Tolerance in Rice
Source: Rice (N Y). 2023 Apr 21;16:21. doi: 10.1186/s12284-023-00637-0 (PMC10121980; doi:10.1186/s12284-023-00637-0)
Supplement: Supplementary file 2 — Additional file 2. Table S1: List of primers used in this study. Table S2: Differential expression data of stress-related in the STK-OE plants. [file 12284_2023_637_MOESM2_ESM.docx]

**Additional information**

**Table S1. List of primers used in this study**

| Primer | Sequence (5'–3') |
| --- | --- |
| STK-qPCR-F | TTGTGGAGGTGCTGATGCTG |
| STK-qPCR-R | GAGTGGTCCAGTCCAGAGCC |
| STK-YFP-F | CGGGATCCATGGGCATCTTCTGCTGCTTCCAGT |
| STK-YPF-R | CGGAATTCTGCGGCGGGGCGTTGGTC |
| STK-Cas9-CX-F | TCCAGTCCGAGGACAGAGG |
| STK-Cas9-CX-R | GGCTCAGCATCAGCACCTC |
| 03g17480-qPCR-F | GCTGAGACGGTTGCTGCTCT |
| 03g17480-qPCR-R | GCTTCGATTCTGTTGATTTCCTC |
| 10g38140-qPCR-F | GAGACGGTGGGGAAGAAGAG |
| 10g38140-qPCR-R | CCCGAAGCCCTGGTAGAAT |
| 10g38710-qPCR-F | GTCCCCGTGCTCATCCAC |
| 10g38710-qPCR-R | GAACGCCAGCCATTCCAG |
| OsABAR1-qPCR-F | GAGGAAGTCAAGCATCAACGG |
| OsABAR1-qPCR-R | ACCCAGTGGATAACCTGACCCT |
| Os3BGlu6-qPCR-F | ACACCTTTGGAAAGATCACCGAC |
| Os3BGlu6-qPCR-R | GAGGTCCCAGTGGTAGAGTGTCA |
| OSBZ8-qPCR-F | ATCCAGCCCTTAGTCCATACCTT |
| OSBZ8-qPCR-R | AGGACCCAAGATAAATGGAGGAG |
| OsSIK1-qPCR-F | GGGGACTGCATCTCCTTAAAATA |
| OsSIK1-qPCR-R | GGACAATGTGGAAGGGATGG |
| OsSAPK10-qPCR-F | GACATGCCGATAATGCACGA |
| OsSAPK10-qPCR-R | CTCCTTGAAGCGGATGATGTT |

**Table S2. Differential expression data of stress-related in the *STK*-OE plants**

| Gene ID | Gene Name | WT readcount | OE5 readcount | Up/Down-Regulation (OE5/WT) | P-value | log2FoldChange (OE5/WT) |
| --- | --- | --- | --- | --- | --- | --- |
| LOC_Os03g17480 | / | 35.45 | 291.40 | Up | 0.011152 | 3.0386 |
| LOC_Os10g38140 | / | 1336.87 | 3546.98 | Up | 0.034051 | 1.4077 |
| LOC_Os10g38710 | / | 10669.57 | 25752.55 | Up | 0.024579 | 1.2712 |
| LOC_Os04g44500 | *OsABAR1* | 533.75 | 1323.45 | Up | 0.026026 | 1.3099 |
| LOC_Os03g11420 | *Os3BGlu6* | 17.61 | 50.00 | Up | 0.036037 | 1.5019 |
| LOC_Os01g46970 | *OSBZ8* | 438.51 | 1093.14 | Up | 0.0034216 | 1.3179 |
| LOC_Os06g03970 | *OsSIK1* | 31.03 | 119.26 | Up | 0.033263 | 1.9412 |
| LOC_Os03g41460 | *OsSAPK10* | 413.67 | 1471.46 | Up | 0.00081767 | 1.8307 |
